# Supplementary material for: Enhancements in yield, agronomic, and zinc recovery efficiencies of rice-wheat system through bioactive zinc coated urea application in Aridisols
Source: PLoS One. 2023 Mar 9;18(3):e0282615. doi: 10.1371/journal.pone.0282615 (PMC9997952; doi:10.1371/journal.pone.0282615)
Supplement: Attachment — Submitted filename: Response to Reviewers.docx [file pone.0282615.s002.docx]

**Editors Comments**

1. A rebuttal letter that responds to each point raised by the academic editor and reviewer(s). You should upload this letter as a separate file labeled 'Response to Reviewers'.

Response: A separate file labelled 'Response to Reviewers' was uploaded

2. A marked-up copy of your manuscript that highlights changes made to the original version. You should upload this as a separate file labeled 'Revised Manuscript with Track Changes'.

Response: A separate file labelled 'Revised Manuscript with Track Changes' was uploaded

3. An unmarked version of your revised paper without tracked changes. You should upload this as a separate file labeled 'Manuscript'.

Response: An unmarked version of revised paper (without tracked changes) was uploaded as a separate file labelled 'Manuscript'.

**Journal Requirements**

Please review your reference list to ensure that it is complete and correct. If you have cited papers that have been retracted, please include the rationale for doing so in the manuscript text, or remove these references and replace them with relevant current references. Any changes to the reference list should be mentioned in the rebuttal letter that accompanies your revised manuscript. If you need to cite a retracted article, indicate the article’s retracted status in the References list and also include a citation and full reference for the retraction notice.

**Response:** Reference list has been corrected and is complete now (Line # 284, 323)

**Review comments to Author**

**Reviewer 1**

No Response

**Reviewer 2**

The authors have addressed almost all the concerns. The authors are advised to make minor typographical and English language related corrections. The manuscript may be accepted now.

**Response:** As suggested, typographical and English language related corrections have been made throughout the manuscript.
